# GACDN: generative adversarial feature completion and diagnosis network for COVID-19

**DOI:** 10.1186/s12880-021-00681-6

**Published:** 2021-10-21

**Authors:** Qi Zhu, Haizhou Ye, Liang Sun, Zhongnian Li, Ran Wang, Feng Shi, Dinggang Shen, Daoqiang Zhang

**Affiliations:** 1 College of Computer Science and Technology, Nanjing University of Aeronautics and Astronautics, Nanjing, 211106 China; 2 Corroborative Innovation Center of Novel Software Technology and Industrialization, Nanjing, 210093 China; 3 School of Biomedical Engineering, ShanghaiTech University, Shanghai, China; 4 Department of Research and Development, Shanghai United Imaging Intelligence Co., Ltd., Shanghai, 201807 China

**Keywords:** Chest computed tomography, COVID-19, GAN, Incomplete multi-view

## Abstract

**Background:**

The outbreak of coronavirus disease 2019 (COVID-19) causes tens of million infection world-wide. Many machine learning methods have been proposed for the computer-aided diagnosis between COVID-19 and community-acquired pneumonia (CAP) from chest computed tomography (CT) images. Most of these methods utilized the location-specific handcrafted features based on the segmentation results to improve the diagnose performance. However, the prerequisite segmentation step is time-consuming and needs the intervention by lots of expert radiologists, which cannot be achieved in the areas with limited medical resources.

**Methods:**

We propose a generative adversarial feature completion and diagnosis network (GACDN) that simultaneously generates handcrafted features by radiomic counterparts and makes accurate diagnoses based on both original and generated features. Specifically, we first calculate the radiomic features from the CT images. Then, in order to fast obtain the location-specific handcrafted features, we use the proposed GACDN to generate them by its corresponding radiomic features. Finally, we use both radiomic features and location-specific handcrafted features for COVID-19 diagnosis.

**Results:**

For the performance of our generated location-specific handcrafted features, the results of four basic classifiers show that it has an average of 3.21% increase in diagnoses accuracy. Besides, the experimental results on COVID-19 dataset show that our proposed method achieved superior performance in COVID-19 vs. community acquired pneumonia (CAP) classification compared with the state-of-the-art methods.

**Conclusions:**

The proposed method significantly improves the diagnoses accuracy of COVID-19 vs. CAP in the condition of incomplete location-specific handcrafted features. Besides, it is also applicable in some regions lacking of expert radiologists and high-performance computing resources.

## Introduction

In December 2019, a novel coronavirus was recognized in Wuhan, China. It was later named as coronavirus disease 2019 (COVID-19). Until now, it has spread all over the world, infecting millions of people and causing more than 3,170,000 death [1–6].Community-acquired pneumonia (CAP) presents symptoms of respiratory infection (such as cough and fever) that are indistinguishable from COVID-19. Causes of CAP include a number of bacterial and viral infections. With the introduction of pneumococcal conjugate vaccines, viruses are an increasingly common cause of CAP. As a type of pneumonia, in clinical diagnosis, the manifestations of COVID-19 and CAP share high degree of similarity in chest computed tomography (CT) images to some extent. There is an urgent need for rapid and effective computer-assisted diagnosis methods to improve the diagnosis accuracy.

At present, main diagnostic methods for COVID-19 are real-time reverse transcriptase polymerase chain reaction (RT-PCR) and CT [7–9]. RT-PCR test requires repeat checking to reduce misdiagnosis and missed diagnosis, especially in the early days of the outbreak. Chest CT image is easily accessible in most hospitals and more intuitive to observe the manifestations and severity of lesions, which is conducive to further diagnosis and treatment. CT findings of COVID-19 include multiple patchy ground-glass opacity and consolidation on both lungs, mostly distributed along the bronchial vascular bundle and subpleural. Thickened blood vessel shadows could be seen between them, which appear as fine grid-like shadows, showing the crazy-paving pattern [10, 11]. These imaging findings can be used to distinguish COVID-19 from CAP by artificial intelligence algorithms, assisting clinicians and radiologists in the diagnosis.

**Fig. 1:**
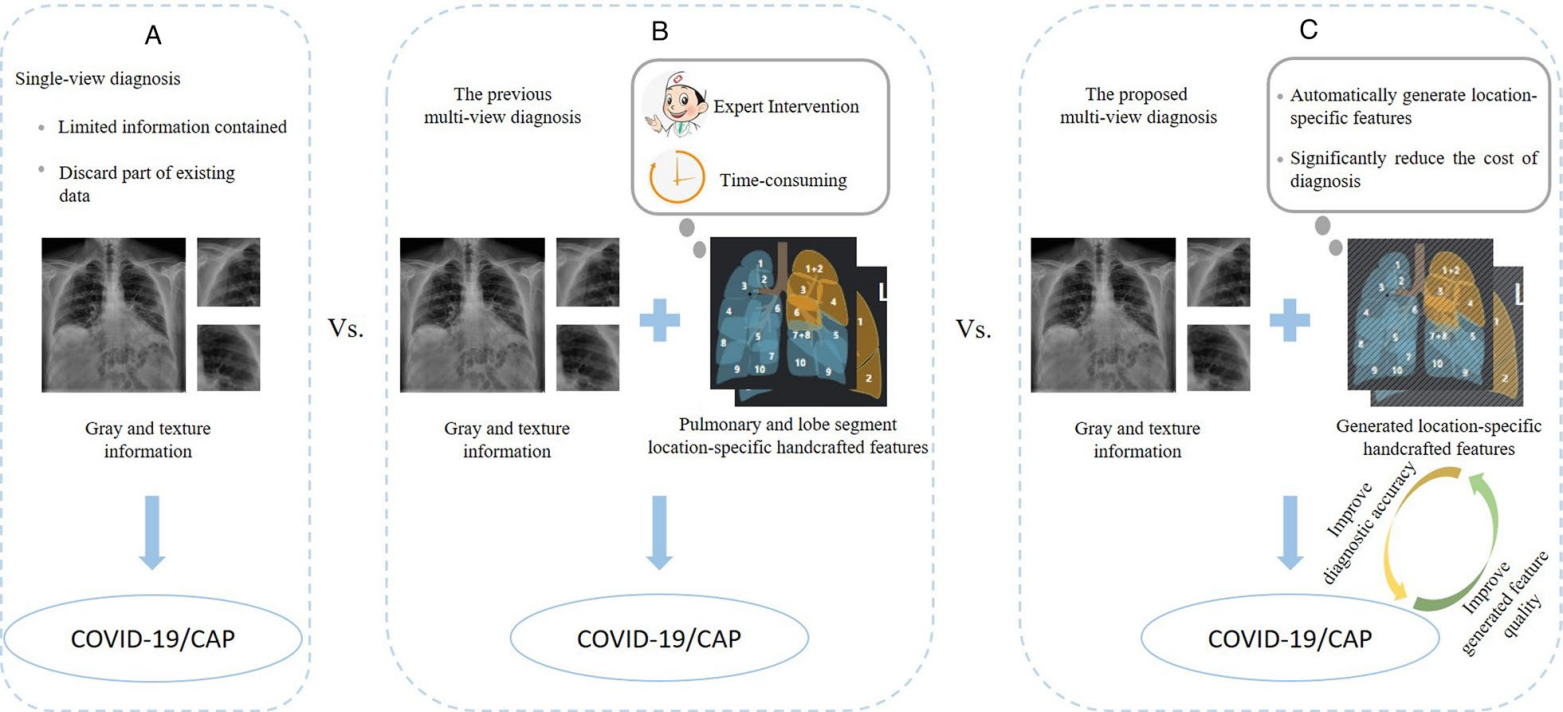
Illustration of three frameworks. **a** Single-view diagnosis. **b** Original multi-view diagnosis by segmenting CT images for location-specific features. **c** Our proposed GACDN for simultaneously generating handcrafted features and making accurate diagnoses

Recently, many machine learning algorithms have achieved promising results in diagnose task [12–15]. Except for the framework of single-view diagnosis shown in Fig. 1a, most of them use the multi-view diagnosis based method in Fig. 1b. These methods use both radiomic and handcrafted features in the diagnosis task and a number of studies have shown that these different views do have a mutual promotion in classification performance [16]. In practice, the fully manual delineation of lung structure for each subject often takes 1–5 h. Later, the time will be shortened to 4 min by algorithm [17]. However, this approach still has several disadvantages: (1) As the number of suspected and infected patients rises, the overall time required to extract location-specific features accumulates rapidly. (2) The extraction algorithm may be unable to perform without expert radiologists and high-performance computing resources in underdeveloped areas [18]. (3) It can only obtain the segmented results, which means other algorithms are still needed to intervene in the diagnosis [19]. Relatively, the radiomic features directly obtained from original CT images by calculating the parameter matrices of the image are easy to get, exploring its underlying information comprehensively is more practical for future studies. If handcrafted features can be estimated according to CT images without segmentation or radiomic features, it will greatly assist clinical diagnosis, but there is a lack of such research.

To address this issue, as shown in Fig. 1c, we propose the framework of incomplete multi-view diagnosis based on the generated handcrafted features. Compared to Fig. 1a, our method handle the problem that the single-view diagnosis framework ignored the underlying information in location-specific handcrafted features. Compared to Fig. 1b, by making full use of radiomic and existing acquired handcrafted features, the advantages of multi-view diagnosis in Fig. 1b are retained while the disadvantages of getting handcrafted features mentioned above are overcame in our proposed method.

**Fig. 2:**
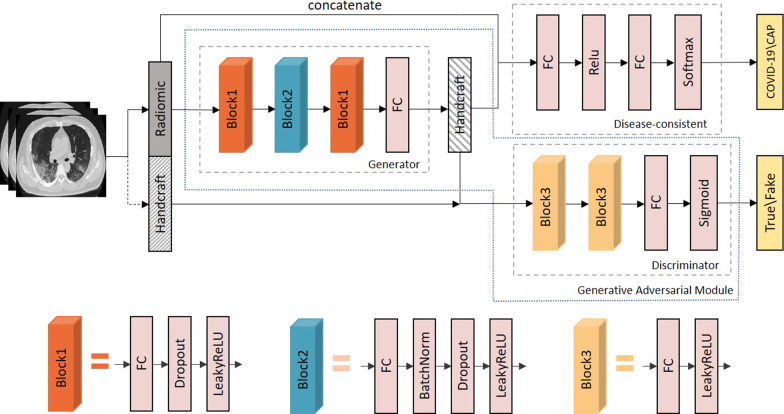
Illustration of the framework of the proposed method—generative adversarial feature completion and diagnosis network (GACDN), including three networks. All networks are upgraded by back propagation

As shown in Fig. 2, the proposed GACDN consists of three networks, including generator, discriminator and the disease-consistent network. Specifically, the generator synthesizes location-specific features, and the discriminator measures the quality of them. Meanwhile, to keep the diagnose results of generated features consistent with completely real samples, the disease-consistent network is employed for COVID-19 diagnosis based on the real radiomic features and completed location-specific features. Disease information of each patient would participate in the training which guarantees that the generated location-specific features would not deviate in label space. Besides, high quality of generated features would also improve the diagnoses accuracy.

In brief, the major contributions of this paper are fourfold.

1. To address the problem of the high cost of acquiring handcrafted features, we propose a generative adversarial feature completion and diagnosis network.
2. The proposed GACDN simultaneously generates handcrafted features by radiomic features and makes accurate diagnoses based on both original and generated features.
3. We apply the disease-consistent network combining with GAN to ensure the mutual promotion of the diagnoses accuracy and the quality of generated features.
4. Extensive experiments show that our proposed method can achieve better performance. More importantly, reducing technical and time cost, ensuring the accuracy, sensitivity and specificity compared with results of the state-of-the-art methods.

The remainder of this paper is organized as follows: we briefly review GAN and incomplete multi-view tasks in “Review of literature” section. In “Methods” section, we present our GACDN modal. In “Results” section, we describe the experiment settings and compare our method with state-of-the-art related approaches. In “Discussion” section, we make a discussion about the results. Finally, the conclusion is provided in “Conclusion” section.

## Review of literature

### Generative adversarial networks (GAN)

In recent years, generative adversarial networks have shown promising performance in generating high quality images [20–22]. The original GAN architecture contains a generative model *G* that seizes the distribution of training data to imitate them and a discriminative model *D* reckons the probability that an input sample came from *G* or training set. Both of them are defined by multilayer perceptrons and can be trained by back propagation until getting the solution that *G* recovering the original data distribution and *D* equal to 1/2 every time.

Due to the excellent performance, GAN is used in many fields.

Conditional generative adversarial network (CGAN) achieves good performance [23] in image synthesis task. Improved by CGAN, the label information is added to both *G* and *D*, which is proved to be useful and become a common practice to promote the performance of networks to translate input images into output images. The Pix2Pix model provides a universal solution to this task, in which an $L_{1}$ norm is used to constrain the difference between real and generated images [24]besides the adversarial loss. In the framework of the Laplacian pyramid, the LAPGAN adopt a cascade of convolutional neural networks to synthesize images in a coarse-to-fine fashion [25].

In super-resolution field [26–28], the low-resolution image is translated to a high-resolution image with the trained model inferring real details of the low-quality areas during up-sampling.

In the field of medical imaging, there are commonly two aspects GAN are utilized. The first is extracting the underlying information of the training data for generating new images. This property makes GANs effective in dealing with data incompletion and patient privacy problems. The second is improving the discriminative ability of the classifier, where the discriminator can be used in classification tasks or diagnosis after training. In [29], Liu has proposed a disease-image specific deep learning framework. They synthesized the missing MRI/PET image of a patient by its existing PET/MRI image. In [30], Nie has proposed a framework which adopted a binary cross entropy loss and $L_{2}$ loss for the generator to synthesis CT images from MRI images. In addition, they embed an image gradient difference loss into the training procedure to estimate the difference between real and fake CT images. The estimated error is used to maintain regions with strong gradients (such as edges) to effectively compensate for the $L_{2}$ loss.

### Incomplete multi-view tasks

In many applications, multi-view data will be generated and used. For pneumonia, CT or X-ray test would be performed to diagnose. For COVID-19, RT-PCR, CT and X-ray are three different ways to diagnose. These different test indicators can be seen as different views, they have different preferences and functions. Combination of these results can be more comprehensive to evaluate the course of a patient. However, not all patients have a complete examination report, they would give up a certain examination due to its expensive fee or the examination results were lost. These circumstances result in the prevalence of incomplete multi-view data.

Recently, various methods for incomplete multi-view have been proposed. In [31], an incomplete multi-view clustering algorithm based on non-negative matrix (NMF) decomposition is proposed, which utilizes NMF to map various view to the hidden space, and uses co-regularized term to make the representation of the hidden space of various modes more uniform. In [32], the algorithm is optimized by adding $L_{2,1}$ regular terms to align hidden space, which is a double alignment. These two methods process the missing values and do not discard incomplete samples either. A hidden space is created to explore the comprehensive information of available data. However, a large amount of useful information is often lost along with the missing of data. Then, more effective methods can be used to complete the incomplete data by filling in the missing values to elevate the performance of the following tasks. In [33], On the basis of the common representation obtained by consensus representation learning, reverse graph regularization is added, and manifold learning is used to predict the missing values, so as to form a complete framework. These two parts are iteratively optimized to further explore the missing data.

## Methods

In this paper, we proposed a generative adversarial feature completion and diagnosis network to simultaneously generate handcrafted features by radiomic features and make accurate diagnoses based on both original and generated features for COVID-19. In this section, we introduce the generative adversarial module and disease-consistent network in our proposed GACDN.

Briefly, in our proposed method, given the training set that $\left\{ X^{n},y^{n}\right\} ^{N}_{n=1}$, where $X^{n}=[x^{n}_{R},x^{n}_{M}]$ is a complete sample and $y^{n}$ is the corresponding disease label (i.e., $y^{n}=0$ is CAP and $y^{n}=1$ is COVID-19), *N* is the number of all samples. $x^{n}_{R}\in \mathbb {R}^{m_{1}*1}$ is the radiomic feature of CT image of sample *n*, $x^{n}_{M}\in \mathbb {R}^{m_{2}*1}$ is the handcrafted feature and $m_{1}$ and $m_{2}$ are the dimensions of radiomic and handcrafted features respectively. The function of generator *G* to be learned is: $f(G): x^{n}_{R}\rightarrow {\hat{x}}^{n}_{M}$, ${\hat{x}}^{n}_{M}$ is the generated handcrafted feature and the function of discriminator *D* is to be learned is: $f(D): {x}^{n}_{M}/{\hat{x}}^{n}_{M}\rightarrow 1/0$. In the end, the classifier *C* is adopted to make the final diagnosis.

### Generative adversarial module

As shown in Fig. 2, the proposed generative adversarial module consists of the generator to generate the location-specific features, and the discriminator to estimate the generated sample.

The generator *G* inputs $x_{R}$, the original radiomic features and outputs ${\hat{x}}_{M}$ as the generated location-specific handcrafted counterpart. The discriminator *D* predicts value of the real sample to be 1 and the value of the generated sample to be 0, maximizing the difference value between the output results of the real and fake samples. The function of *D* is as follows:

1 $$f_{D}(x_{i})= {\left\{ \begin{array}{ll} 1, &{\quad} x_{i}\in X_{M},\\ \\ 0, &{\quad} x_{i}\in {\hat{X}}_{M}. \end{array}\right. }$$

In original GAN, adversarial loss between generator and discriminator is used for training. We preserve this architecture as the foundation in order to improve the authenticity of generated samples synthesized by the generator and the capability of discriminator to distinguish real samples from fake ones. Binary cross entropy (BCE) loss is adopted to realize the adversarial training between generator and discriminator. The adversarial loss is as follows:

2 $$\begin{aligned} L_{adversarial}&=\mathbb {E}_{x_{M}\in X_{M}}log\Big (D(x_{M})\Big ) \\&\quad+\mathbb {E}_{x_{R}\in X_{R}}log\Big (1-D\big (G(x_{R})\big )\Big ) \end{aligned}$$

In GAN, the discriminator tries to distinguish between real and fake location-specific handcrafted features, but the generator seeks to produce them that the discriminator cannot identify. The probability distribution of the original space can be learned by the basic generative adversarial networks, so that the generated location-specific handcrafted features conform to the probability distribution of the original space. However, it only ensures that the generated handcrafted features have the same distribution as the existing ones to some extent. The weakness is lacking of stability in training procedure and the principle of one-to-one correspondence—the generated location-specific handcrafted features should correspond with its radiomic counterparts. These features are interrelated and also independent of each other. The generation process of the generator cannot be well constrained by adversarial loss because of the vanishing gradient or exploding gradient problem [34, 35].

To address this issue, we add the original handcrafted features corresponding to the input radiomic features as label into training process of the generator *G* and constrain it by mean square error (MSE). Such a constraint can well reduce the error between generated data and the real data. Besides, similarity between them would be maximized at a very low cost at the same time. The MSE loss is as follows:

3 $$L_{mse}=\Big \Vert x_{M}-G(x_{R})\Big \Vert _{2}^{2}$$

### Disease-consistent network

In our method, the input features are already extracted from the CT images so the extra extraction operation is unnecessary. In addition, the labels of all training data are complete. Based on this, we introduced a disease-consistent network, *C*, to identify whether the patient belongs to COVID-19 or CAP based on the input data. In the process of training *C*, the generator is also updated through back propagation. By this means, the produce of generator can be further promoted and constrained from the most intuitive perspective to avoid the occurrence of overfitting. However, the distribution drift will inevitably occur between the generated data and the real data. The disease-consistent network trained by the real data usually performs poorly in the generated samples, which is due to the occurrence of the distribution drift. To solve this problem, we do not simply use completely original or generated features to train *C* which can lead to the result of the test samples totally invalid. We combined the real radiomic features and the generated location-specific handcrafted counterparts as an input to train the disease-consistent network. After that, it can effectively avoid the distribution drift and improve the disease diagnosis accuracy. Also, the trained *C* can be directly used in the diagnosis of follow-up work.

Formally, the loss function of *C* is as follows:

4 $$L_{consistent}=\Big \Vert y-C\big (x_{R},G(x_{R})\big )\Big \Vert _{2}^{2}$$

At last, we adopt two adjustable weights to balance the effect of each part. The overall loss is as follows:

5 $$\begin{aligned} {L}(x_{R},x_{M},y;G,D,C)&= \mathbb {E}_{x_{M}\in X_{M}}log\Big (D(x_{M})\Big ) \\ &\quad+ \mathbb {E}_{x_{R}\in X_{R}}log\Big (1-D\big (G(x_{R})\big )\Big ) \\ &\quad+\lambda _{1}\Big \Vert x_{M}-G(x_{R})\Big \Vert _{2}^{2} \\ &\quad+ \lambda _{2}\Big \Vert y-C\big (x_{R},G(x_{R})\big )\Big \Vert _{2}^{2} \end{aligned}$$

### Description of datasets

The dataset used is from our previous studies [13], which contains features from a total of 2522 CT images. Among them, 1,495 cases are confirmed to be positive for COVID-19 by RT-PCR and are obtained from January 9, 2020 to February 14, 2020. The remaining 1027 samples are patients with CAP, and these images are obtained between July 30, 2018 and February 22, 2020. The information of these 2522 cases are summarized in Table 1.

**Table 1:** Information of 2522 chest CT scans from COVID-19 dataset

TYPE	Male	Female	Total
COVID-19	770	725	1495
CAP	488	539	1027
Total	1258	1264	2522

All patients underwent thin-section CT scans of the chest. Specifically, CT scanners include uCT 780 from UIH, Optima CT520, Discovery CT750, LightSpeed 16 from GE, Aquilion ONE from Toshiba, SOMATOM Force from Siemens, and SCENARIA from Hitachi. CT protocol includes: 120 kV, reconstructed slice thickness ranging from 0.625 to 2 mm, with breath hold at full inspiration.

#### Data preprocessing

First, we calculated the radiomic features based on the original CT images: Gray features are composed of first-order statistics that describe the voxel intensity distribution in CT image, such as the maximum, minimum; Texture features are composed of gray level co-occurrence matrix (GLCM), gray level size zone matrix (GLSZM), gray level run length matrix (GLRLM), neighboring gray tone difference matrix (NGTDM) and gray level dependence matrix (GLDM). In the end, a total of 93 radimoic features were obtained.

Then, we adopt the method proposed in [17] to segment the image through VB-NET for the lesion area and the lung fields. The segment of lesion area is mainly based on some clinical manifestations of COVID-19 and CAP. Such as ground glass opacity and thickened blood vessel shadows. For these lesions, we calculated their volume, number of lesions, histogram distribution, and surface area. At the same time, the image was also divided into left and right lungs, five lobules, and 18 pulmonary segments according to the structure of human lungs. In the end, a total of 96 location-specific handcrafted features were obtained. Total of 189 features are shown in Table 2.

**Table 2:** Summary of different types of features

	Features	Numbers	Total
Radiomic	Gray	19	93
	Texture	74	
Handcrafted	Histogram	30	96
	Number	24	
	Intensity	2	
	Surface	7	
	Volume	33	

#### Incomplete data

Previous studies have shown that both radiomic features and handcrafted features can achieve good classification results. The accuracy of the former can reach 87.6% and the latter has an accuracy of 89.41%. When we combine two of them, the precision of NN can be improved significantly. Therefore, we believe that the two perspectives of data contain the information of the original image more comprehensively. However, in practice, it is very difficult to acquire handcrafted features: First, it takes a lot of time to segment CT images. In such an emergency of epidemic situation, rapid and accurate is the primary requirement, which cannot meet one of the requirements. Second, segmentation algorithms need the support of high-performance computers, which are not available in some areas. Third, this segmentation algorithm is internal and not easy to obtain. Based on the existing data, we assume that the training set contains all the data of the two views and the test set contains only the easily acquired radiomic features.

### Experiment settings

Based on the dataset and problems to be solved, we designed a three-part experiment to verify the effectiveness of our method. First part is the improvement of the diagnoses accuracy. We adopt the basic classifiers to compare the performance of the radiomic data only and complete data among which the handcrafted features are generated by our framework, respectively.

Second part is to compare with other feature extraction method. Feature extraction and data generation methods would solve the practical problem from two different perspectives. Both of them would improve accuracy. However, the research results of CT image segmentation to collect handcrafted features and the information contained in existing handcrafted features are discarded if we implement researches on radiomic data only. Our approach would reduce the requirements for medical sources while making the best of existing research to improve the diagnosis. The methods we choose are summarized as follows:1)Low-rank representation (LRR) [36], 2)Latent low-rank representation (LatLRR) [37], 3)Locality preserving projections (LPP) [38], 4)Stacked autoencoder (SAE) [39], 5)Adaptive feature selection deep forest(AFS-DF) [13].

Last part is a comparison with the state-of-art multi-view incomplete recovery methods. The methods we choose are as follows: (1) partial multi-view clustering (PVC) [40], (2) unified embedding alignment framework (UEAF) [33], (3) incomplete multi-view clustering via graph regularized matrix factorization (IMC-GRMF) [41], (4) adaptive graph completion based incomplete multi-view clustering (AGC-IMC) [42], (5) generative adversarial incomplete network (GAIN) [43].

The data set is randomly divided into 90% training set and 10% test set, and tenfold cross-validation is used to adjust the parameters. Diagnostic performance is evaluated in terms of accuracy (ACC), sensitivity (SEN), specificity (SPE), recall (REC), precision (PRE) and F1-score (F1).

## Results

In the first part, we perform the basic classifiers on radiomic only data and completed data in which the missing values filling by our proposed algorithm. The effect of our proposed method is shown in Table 3. Specifically, for support vector machine (SVM), we add Gaussian kernel and use grid search method for parameter optimization. For K-nearest neighbor (KNN), we choose Euclidean distance to measure the similarity. In neural network (NN), we adopt a fully connected network and back propagation to update parameters for training. As the Table 3 shows, the accession of generated features significantly improves the diagnostic performance. For accuracy, four classifiers improve 3.09%, 2.53%, 3.84% and 3.04% respectively, with an average increase of 3.21%. The neural network has achieved best results both before and after. In terms of SPE, the performance of some of these classifiers is not all elevated or even slightly reduced, but all of them have different degrees of improvement in AUC. For SEN, our processed data improves about 2.79% over radiomic only with the same fold assignments. It is worth nothing that COVID-19 is a highly contagious disease, SEN represents the probability of a true positive diagnosis, which is of a great significant for the evaluation of a model. The improvement is due to the fact that the combination of two perspectives of data does contain more comprehensive information than the separate radiomic data. At this point, we have reasons to believe that generated handcrafted features are able to effectively improve the diagnoses accuracy.

**Table 3:** Effects of data generated by GACDN With baselines

	ACC (%)	SEN (%)	SPE (%)	AUC (%)
*Radiomic*				
LR	87.74 ± 1.20	90.34 ± 1.86	83.90 ± 3.07	87.12 ± 1.36
SVM	86.59 ± 1.93	89.30 ± 2.64	82.72 ± 3.57	86.01 ± 1.97
KNN	85.76 ± 3.27	90.04 ± 2.19	79.72 ± 6.96	84.88 ± 3.68
NN	87.19 ± 1.25	84.23 ± 2.75	91.60 ± 2.95	87.91 ± 1.22
*Radiomic + GACDN*				
LR	90.83 ± 1.25	92.41 ± 1.86	88.52 ± 2.63	90.46 ± 1.29
SVM	89.13 ± 2.06	90.24 ± 2.77	87.62 ± 3.12	88.93 ± 2.10
KNN	89.61 ± 1.78	91.97 ± 2.54	86.19 ± 2.09	89.08 ± 1.64
NN	90.23 ± 1.43	90.44 ± 1.79	90.98 ± 2.25	91.12 ± 1.36

In the second part, we compare ours with several feature extraction methods. According to the Table 4, traditional methods such as LRR and LPP have a slight improvement in diagnoses accuracy, but the results are slightly worse than those of the deep methods. Improved LatLRR obtains a low-rank representation in hidden space which would reduce the redundancy of features and makes the data easier to be distinguished. Both SAE and AFS-DF belong to the methods based on deep neural network, and both of them adopt nonlinear mapping to extract features. The advantage of our method is that it does not abandon the handcrafted features. The underlying information of existing data is fully explored which would provide more guidance in the diagnosis process and improve diagnoses results.

**Table 4:** Performance comparison with feature selection methods for diagnosis

	ACC (%)	SEN (%)	SPE (%)	AUC (%)
LRR	88.02 ± 1.45	89.02 ± 1.515	86.79 ± 1.83	87.90 ± 1.23
LatLRR	90.43 ± 1.75	90.92 ± 3.175	88.30 ± 3.11	90.11 ± 1.64
LPP	89.12 ± 1.68	90.97 ± 2.862	86.83 ± 2.55	89.08 ± 1.29
SAE	90.20 ± 2.22	91.22 ± 2.662	88.79 ± 3.20	90.01 ± 2.48
AFS-DF	90.67 ± 2.23	90.49 ± 3.010	90.41 ± 3.34	90.91 ± 0.91
GACDN	91.31 ± 1.12	91.62 ± 1.923	91.01 ± 2.31	91.32 ± 1.13

In the last part, considering incomplete multi-view task, we compare our method with a variety of state-of-art methods of incomplete multi-view clustering (IMC). After the handcrafted features predicted by the algorithm, to ensure fairness, the label information is added to the data and train the classifier. The results of different methods are shown in Table 5.

**Table 5:** Performance comparison with incomplete multi-view methods for diagnosis

	ACC (%)	SEN (%)	SPE (%)	AUC (%)
PVC	73.16 ± 5.71	87.89 ± 3.80	51.443 ± 1.53	69.67 ± 3.12
UEAF	88.61 ± 2.00	88.71 ± 2.42	88.55 ± 4.24	88.63 ± 2.24
IMC-GRMF	85.85 ± 2.77	88.59 ± 3.39	81.96 ± 2.91	85.27 ± 2.899
GAIN	88.36 ± 2.44	87.87 ± 2.18	83.838 ± 3.69	86.55 ± 2.68
AGC-IMC	88.69 ± 1.67	89.62 ± 2.56	87.34 ± 1.35	88.48 ± 1.79
GACDN	91.31 ± 1.12	91.62 ± 1.92	91.01 ± 2.31	91.32 ± 1.13

Specifically, with the exception of GAIN, all the other methods are unsupervised algorithms based on matrix decomposition. Admittedly, incomplete multi-view tasks are now mostly studied on unsupervised clustering tasks, and have not been well explored in supervised tasks. Due to the lack of guidance of label information when filling the missing data, the predicted data does not improve the diagnoses accuracy well in this problem. Among them, with respect to ACC, PVC decreases 16% due to the fact that the difference in data distribution of the two views could not be well resolved by common representation; IMC-GRMF decreases 1.538% since the optimization method can only obtain the local optimal solution. However, it shows that UEAF, AGC-IMC, can obtain the structure of samples with complete and incomplete data, so the diagnosis has a certain improvement, but the effect is not obvious. Our method adopts GAN to acquire the data distribution of the original space. In addition, label information is added to generate the missing data according to diseases, which can significantly improve the diagnoses accuracy.

**Fig. 3:**
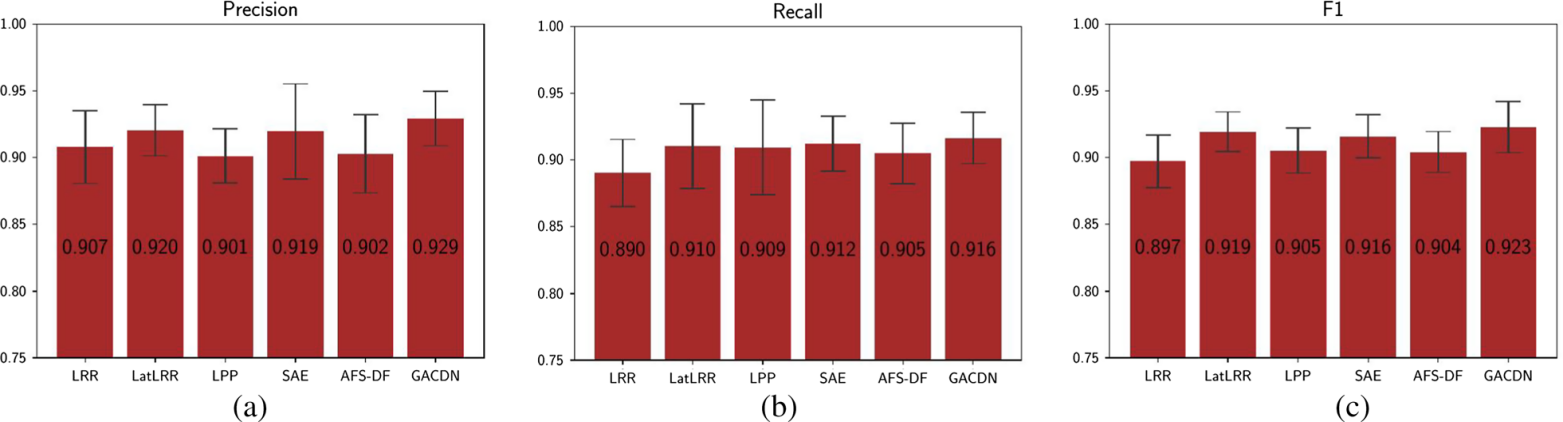
Experimental results with respect to **a** precision, **b** recall and **c** F-score of different feature-selection methods

**Fig. 4:**
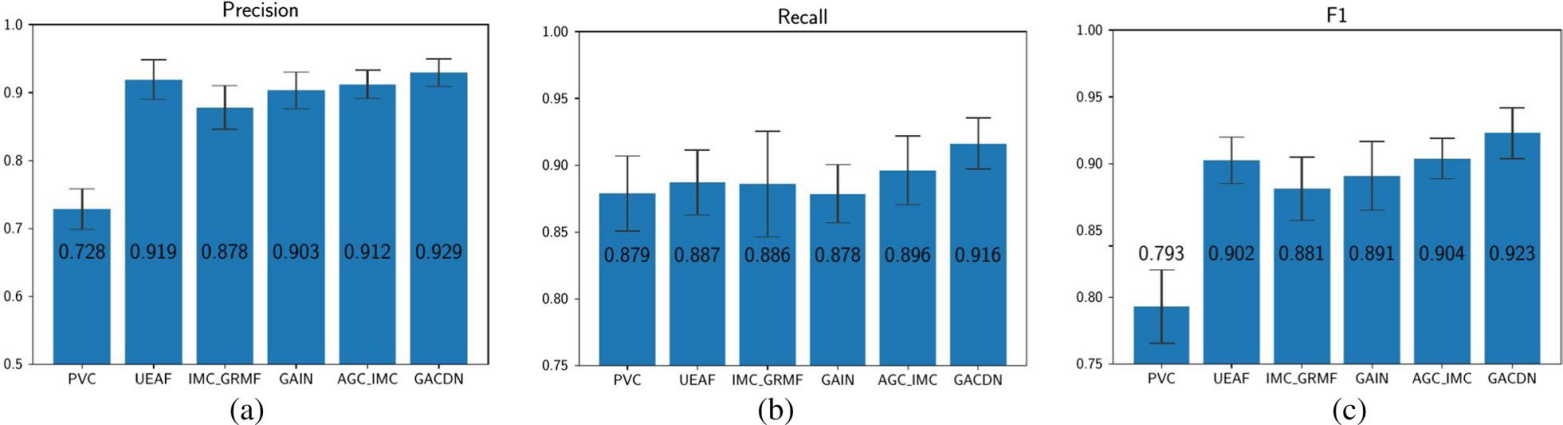
Experimental results with respect to **a** precision, **b** recall and **c** of different incomplete multi-view based methods

**Fig. 5:**
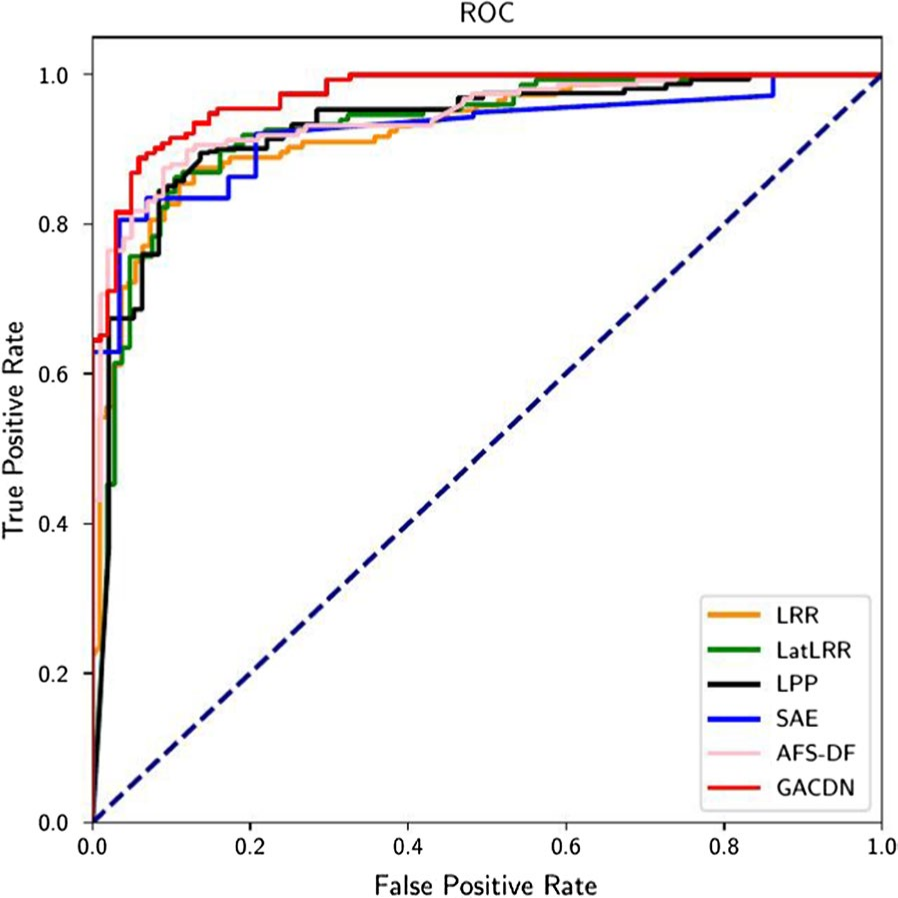
ROC curves achieved by LRR, LatLRR, LPP, SAE, AFS-DF, GACDN in COVID-19 versus CAP classification

**Fig. 6:**
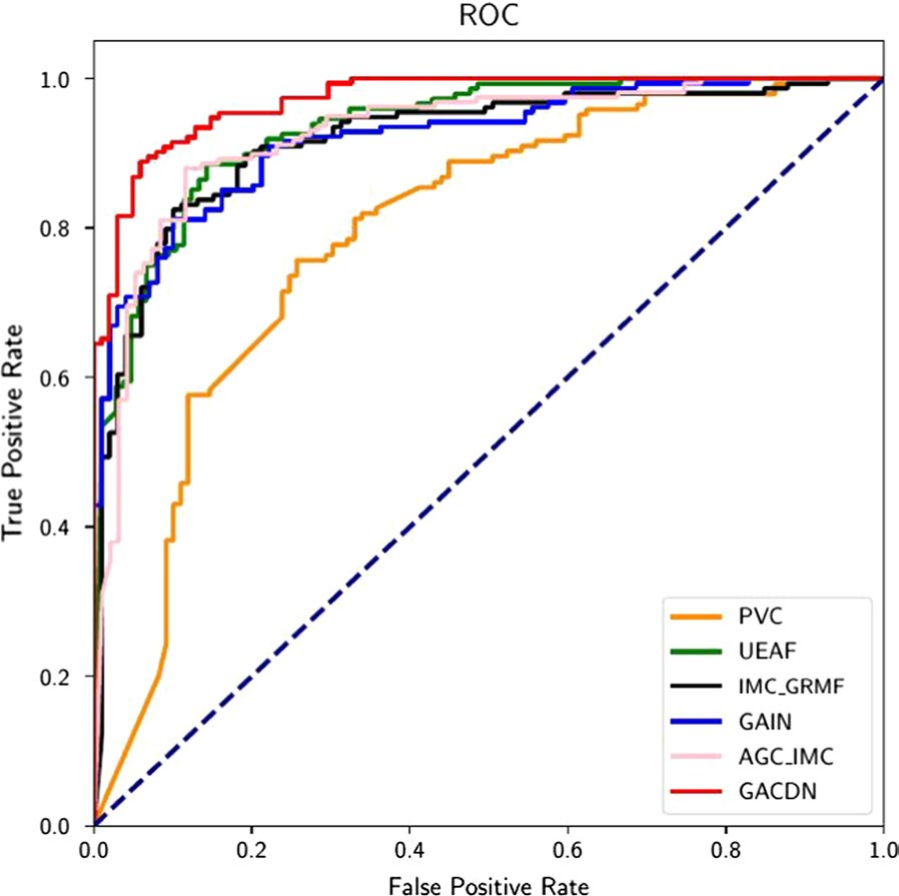
ROC curves achieved by PVC, UEAF, IMC-GRMF, GAIN, AGC-IMC, GACDN in COVID-19 versus CAP classification

Besides, the remaining three indicators are also shown in Figs. 3 and 4. It is obvious that our method can achieve the best results. As shown in Figs. 5 and 6, our proposed GACDN method achieves the best diagnostic performance with the feature extraction and multi-view incomplete based method respectively. These results further validate that integrating auto-generated handcrafted features with radiomic features could be an effective way to handle the practice problem of the high cost of obtaining them and could improve the diagnosis accuracy for COVID-19 vs. CAP.

## Discussion

### Clinical impact

In this subsection, we analyze the clinical impact of the proposed method in three perspectives.

**Fig. 7:**
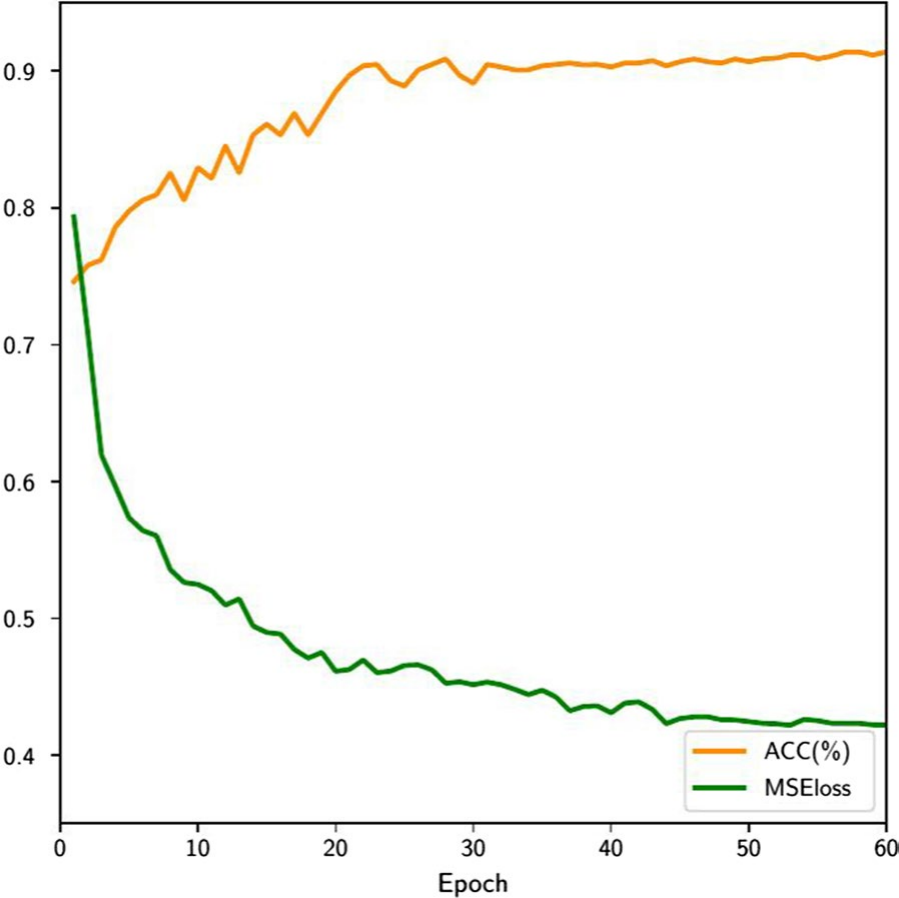
MSEloss and ACC of the test samples as the epoch increases

**Fig. 8:**
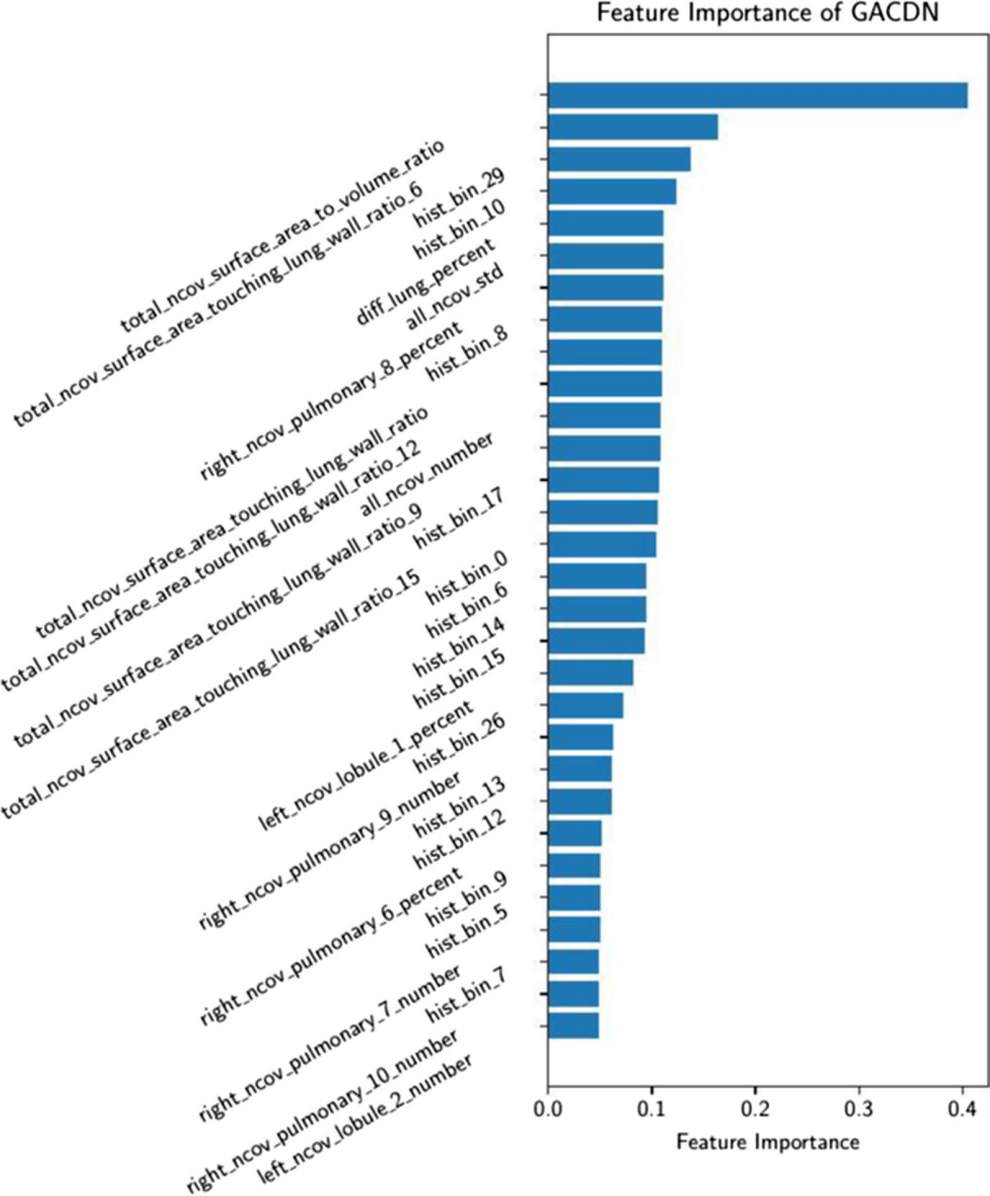
The top 30 important features of handcrafted location-specific features in GACDN

First, we further analyze the relationship between the quality of generated handcrafted features and diagnoses accuracy. As shown in Fig. 7, we calculated the diagnoses accuracy on the test set for each epoch from 0 to 60, as well as the MSEloss of generated features and original features in all test samples. As the generated features are closer to the real ones, the diagnoses accuracy is gradually improved. Since the loss of diagnoses in the training procedure updates the generator with back propagation, it shows that there is a good mutual promotion between the quality of the generated features and the diagnostic accuracy. In the end, the feature importance of handcrafted features is shown in Fig. 8. Among all the handcrafted location-specific features, our method find that the surface area features of lesion area (i.e. surface area to volume ratio, surface area touching lung wall ratio) and gray level histogram of lesion area (i.e. hist bin 29, hist bin 10) are critical which means that these features play an important role in clinical diagnosis.

Second, the method focuses on incomplete multi-modal data fusion for identifying COIVD-19, and achieves promising diagnosis accuracy. If handcrafted features can be estimated according to CT images without segmentation, it will greatly assist clinical diagnosis. The proposed method can be easily implemented by non-AI domain users. It is also applicable in some regions lacking of expert radiologists and high-performance computing resources.

Last, the fully manual segmentation of lung structure for each subject often takes 1–5 h, and it can be shortened to 4 min by algorithm. The radiomic features utilized in our proposed method can be obtained by calculation. Therefore, the proposed method not only reduces the time required for diagnosis, but it also integrates handcrafted feature recovery and diagnosis into a complete framework.

### Parameter sensitivity analysis

In this subsection, the parameter sensitivity of the proposed method is analyzed. There are two parameters to be tuned in problem (5), i.e $\lambda _{1}$ and $\lambda _{2}$. We can directly visualize the results due to the small numbers of parameters. Hence, given a range set from 0.00001 to 10,000, increasing tenfold each time. When a parameter is tuned, the other parameter will be fixed.

**Fig. 9:**
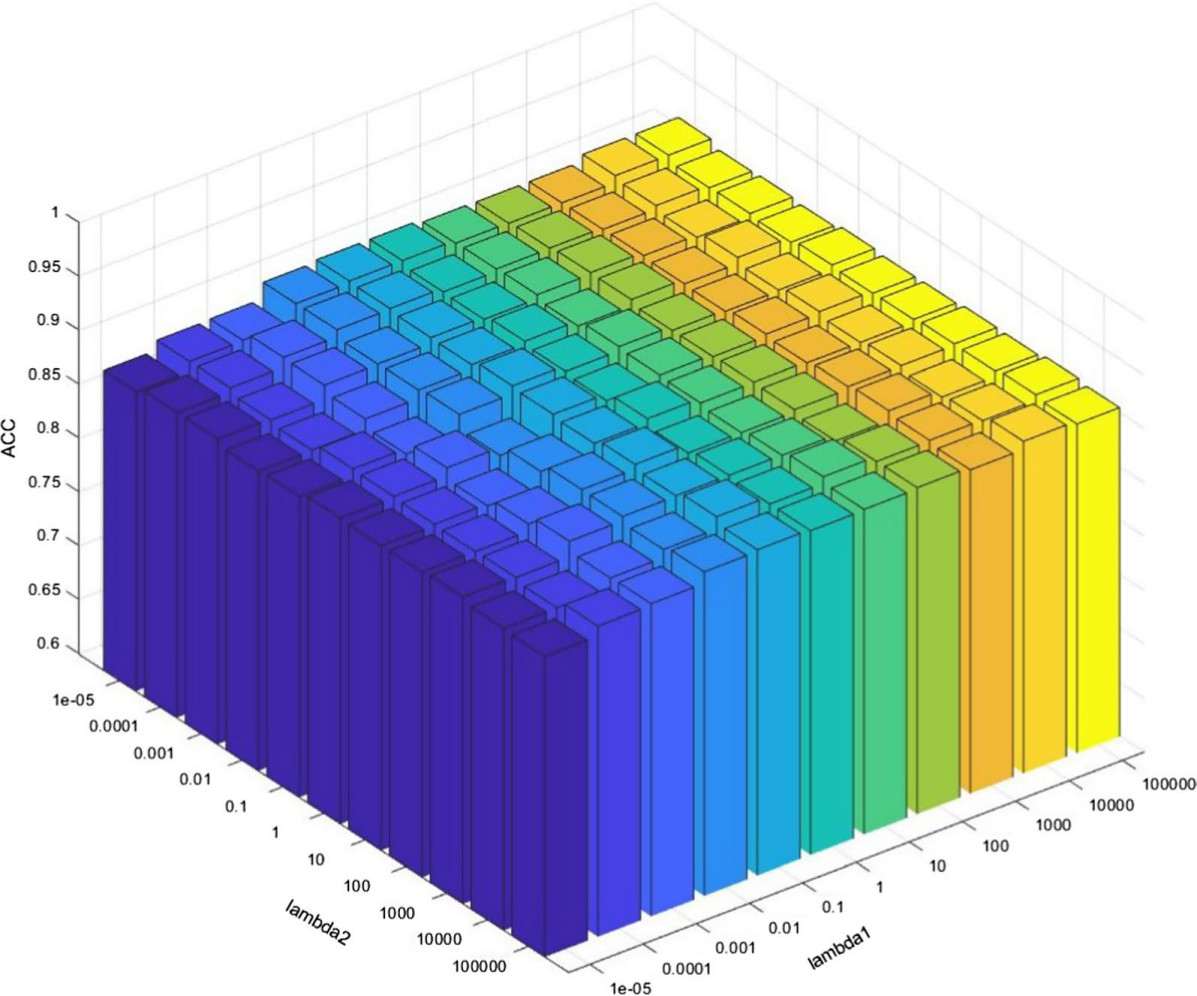
Parameter sensitivity of our proposed method GACDN in COVID-19 versus CAP classification

The result is shown in Fig. 9. As we can see, when both parameters are the minimum value, the worst result is obtained and the predicted special value does not play a good role in improving the classification accuracy. This is mainly because the minimal loss percentage makes the discrimination error unable to play an adequate role in the adversarial training of GAN, resulting in the poor quality of the generated data. Specifically, with the increase of lambda1 before 1, the result has been significantly improved. This is because the increase of the proportion of counter loss makes the training of GAN more stable. For lambda2, the sensitivity is a little smaller than lambda1, which means that the weight of tag information is relatively unimportant as long as it is added. Above all, our method shows robustness to the varying parameters in most cases.

## Conclusion

This paper presents an innovative GAN based model for the problem of missing location-specific handcrafted features in the process of COVID-19 and CAP diagnosis. This method studies the mapping and probability distribution from the radiomic features to handcrafted features on the basis of existing data, and generates handcrafted features according to the prior knowledge. Finally, the model can simultaneously generate location-specific handcrafted features and make accurate diagnosis. Generative adversarial module and disease-consistent network are holistically integrated into one framework in our model. We demonstrate the method significantly improving the diagnoses accuracy without the intervention of experts through a large number of experiments in three aspects: the improvement of baseline classifiers, the comparison with single-view feature extraction methods and the comparison with incomplete multi-view based methods. In addition, our work can be extended from following aspects:

1. The current problem is only for the diagnosis of COVID-19 and CAP. Future work can be expanded to the differential diagnosis tasks of COVID-19, normal and CAP.
2. Our method can only obtain the positive or negative result. Once clinical disease severity data were obtained, multi-task and multi-modal models can be used to predict the presence and severity of disease.

## Data Availability

Not applicable.

## References

[1] Kim H, Hong H, Yoon SH (2020). Diagnostic performance of CT and reverse transcriptase polymerase chain reaction for coronavirus disease 2019: a meta-analysis. Radiology.

[2] Ozturk T, Talo M, Yildirim EA, Baloglu UB, Yildirim O, Acharya UR (2020). Automated detection of COVID-19 cases using deep neural networks with X-ray images. Comput Biol Med.

[3] Wu JT, Leung K, Leung GM (2020). Nowcasting and forecasting the potential domestic and international spread of the 2019-nCoV outbreak originating in Wuhan, China: a modelling study. The Lancet.

[4] Shi H, Han X, Jiang N, Cao Y, Alwalid O, Gu J, Fan Y, Zheng C (2020). Radiological findings from 81 patients with COVID-19 pneumonia in Wuhan, China: a descriptive study. Lancet Infect Dis.

[5] Song F, Shi N, Shan F, Zhang Z, Shen J, Lu H, Ling Y, Jiang Y, Shi Y (2020). Emerging 2019 novel coronavirus (2019-nCoV) pneumonia. Radiology.

[6] Organization WH. Coronavirus disease 2019 (COVID-19): situation report. 2020;82.

[7] Fang Y, Zhang H, Xie J, Lin M, Ying L, Pang P, Ji W (2020). Sensitivity of chest CT for COVID-19: comparison to RT-PCR. Radiology.

[8] Long C, Xu H, Shen Q, Zhang X, Fan B, Wang C, Zeng B, Li Z, Li X, Li H (2020). Diagnosis of the Coronavirus disease (COVID-19): rRT-PCR or CT?. Eur J Radiol.

[9] Li Y, Xia L (2020). Coronavirus disease 2019 (COVID-19): role of chest CT in diagnosis and management. Am J Roentgenol.

[10] Pan Y, Guan H, Zhou S, Wang Y, Li Q, Zhu T, Hu Q, Xia L (2020). Initial CT findings and temporal changes in patients with the novel coronavirus pneumonia (2019-nCoV): a study of 63 patients in Wuhan, China. Eur Radiol.

[11] Kanne JP (2020). Chest CT findings in 2019 novel coronavirus (2019-nCoV) infections from Wuhan, China: key points for the radiologist.

[12] Zheng C, Deng X, Fu Q, Zhou Q, Feng J, Ma H, Liu W, Wang X. Deep learning-based detection for COVID-19 from chest CT using weak label, 2020;1–13 . 10.1101/2020.03.12.20027185.

[13] Sun L, Mo Z, Yan F, Xia L, Shan F, Ding Z, Song B, Gao W, Shao W, Shi F, Yuan H, Jiang H, Wu D, Wei Y, Gao Y, Sui H, Zhang D, Shen D (2020). Adaptive feature selection guided deep forest for COVID-19 classification with chest CT. IEEE J Biomed Health Inform.

[14] Kang H, Xia L, Yan F, Wan Z, Shi F, Yuan H, Jiang H, Wu D, Sui H, Zhang C, Shen D (2020). Diagnosis of coronavirus disease 2019 (COVID-19) with structured latent multi-view representation learning. IEEE Trans Med Imaging.

[15] Zhang J, Gao Y, Gao Y (2016). Detecting anatomical landmarks for fast Alzheimer’s disease diagnosis. IEEE Transa Med Imag.

[16] Shi F, Xia L, Shan F (2021). Large-scale screening to distinguish between COVID-19 and community-acquired pneumonia using infection size-aware classification. Phys Med Biol.

[17] Shan F, Gao Y, Wang J, Shi W, Shi N, Han M, Xue Z, Shi Y. Lung infection quantification of COVID-19 in CT images with deep learning. arXiv preprint arXiv:2003.04655. 2020.

[18] Villalá MAG, Nollen JA, Rico SD, Quiroga GAC, Guirado JLC, De Los Rios GOA (2020). COVID 19, Pathophysiology and prospects for early detection in patients with mild symptoms of the controversial virus in underdeveloped countries. J Health Sci Prev.

[19] Wang L, Shi F, Lin W (2011). Automatic segmentation of neonatal images using convex optimization and coupled level sets. NeuroImage.

[20] Creswell A, White T, Dumoulin V, Arulkumaran K, Sengupta B, Bharath AA (2018). Generative adversarial networks: an overview. IEEE Signal Process Mag.

[21] Goodfellow IJ, Pouget-Abadie J, Mirza M, Xu B, Warde-Farley D, Ozair S, Courville A, Bengio Y. Generative adversarial networks. arXiv preprint arXiv:1406.2661. 2014.

[22] Hong Y, Hwang U, Yoo J, Yoon S (2019). How generative adversarial networks and their variants work: an overview. ACM Comput Surv.

[23] Mirza M, Osindero S. Conditional generative adversarial nets. arXiv preprint arXiv:1411.1784. 2014.

[24] Isola P, Zhu J-Y, Zhou T, Efros AA. Image-to-image translation with conditional adversarial networks. In: Proceedings of the IEEE conference on computer vision and pattern recognition. 2017. p. 1125–34.

[25] Denton E, Chintala S, Szlam A, Fergus R. Deep generative image models using a laplacian pyramid of adversarial networks. arXiv preprint arXiv:1506.05751. 2015.

[26] Chen Y, Shi F, Christodoulou AG, Xie Y, Zhou Z, Li D. Efficient and accurate mri super-resolution using a generative adversarial network and 3d multi-level densely connected network. In: International conference on medical image computing and computer-assisted intervention. Springer; 2018. p. 91–9.

[27] Ledig C, Theis L, Huszár F, Caballero J, Cunningham A, Acosta A, Aitken A, Tejani A, Totz J, Wang Z. Photo-realistic single image super-resolution using a generative adversarial network. In: Proceedings of the IEEE conference on computer vision and pattern recognition, 2017. p. 4681–90.

[28] Sánchez I, Vilaplana V. Brain MRI super-resolution using 3D generative adversarial networks. arXiv preprint arXiv:1812.11440. 2018.

[29] Pan Y, Liu M, Lian C, Xia Y, Shen D. Disease-image specific generative adversarial network for brain disease diagnosis with incomplete multi-modal neuroimages. In: International conference on medical image computing and computer-assisted intervention. Springer; 2019. p. 137–45.

[30] Nie D, Trullo R, Lian J, Petitjean C, Ruan S, Wang Q, Shen D. Medical image synthesis with context-aware generative adversarial networks. Lecture notes in computer science (including subseries Lecture notes in artificial intelligence and Lecture notes in bioinformatics) 10435 LNCS; 2017. p. 417–25 . 10.1007/978-3-319-66179-7_48. arxiv:1612.05362. 10.1007/978-3-319-66179-7_48 PMC6044459 30009283

[31] Shao W, He L, Philip SY. Multiple incomplete views clustering via weighted nonnegative matrix factorization with l2,1 regularization. In: Joint European conference on machine learning and knowledge discovery in databases Springer; 2015. p. 318–34.

[32] Hu M, Chen S. Doubly aligned incomplete multi-view clustering. arXiv preprint arXiv:1903.02785. 2019.

[33] Wen J, Zhang Z, Xu Y, Zhang B, Fei L, Liu H. Unified embedding alignment with missing views inferring for incomplete multi-view clustering. In: Proceedings of the AAAI conference on artificial intelligence, vol. 33; 2019. p . 5393–400.

[34] Hochreiter S (1998). The vanishing gradient problem during learning recurrent neural nets and problem solutions. Int J Uncertain Fuzziness Knowl-Based Syst.

[35] Pascanu R, Mikolov T, Bengio Y. On the difficulty of training recurrent neural networks. In: International conference on machine learning; 2013. p. 1310–8. PMLR.

[36] Liu G, Lin Z, Yu Y, et al. Robust subspace segmentation by low-rank representation. In: ICML; 2010. p. 1, 8. Citeseer.

[37] Liu G, Yan S. Latent low-rank representation for subspace segmentation and feature extraction. In: 2011 International conference on computer vision. IEEE; 2011. p. 1615–22.

[38] He X, Niyogi P (2004). Locality preserving projections. Adv Neural Inf Process Syst.

[39] Masci J, Meier U, Cireşan D, Schmidhuber J. Stacked convolutional auto-encoders for hierarchical feature extraction. In: International conference on artificial neural networks. Springer; 2011. p. 52–9.

[40] Li S-Y, Jiang Y, Zhou Z-H. Partial multi-view clustering. In: Proceedings of the AAAI conference on artificial intelligence. 2014. p. 28.

[41] Wen J, Zhang Z, Xu Y, Zhong Z. Incomplete multi-view clustering via graph regularized matrix factorization. In: Proceedings of the European conference on computer vision (ECCV) workshops. 2018.

[42] Wen J, Yan K, Zhang Z, Xu Y, Wang J, Fei L, Zhang B (2020). Adaptive graph completion based incomplete multi-view clustering. IEEE Trans Multimed..

[43] Yoon J, Jordon J, Van Der Schaar M. GAIN: missing data imputation using generative adversarial nets. In: 35th International conference on machine learning, ICML 2018 13. 2018. p. 9042–51. arxiv:1806.02920.

